# Investigation of Eating Behaviors in Euthyroid Patients With Hashimoto’s Thyroiditis

**DOI:** 10.7759/cureus.81814

**Published:** 2025-04-07

**Authors:** Seher Çetinkaya Altuntaş

**Affiliations:** 1 Endocrinology and Diabetes, Bursa Yuksek Ihtisas Education and Training Hospital, University of Health Sciences, Bursa, TUR

**Keywords:** eating disorders (eds), euthyroid, late-night eating, obesity, hashimoto’s thyroiditis

## Abstract

Background

Hashimoto’s thyroiditis (HT) is an organ-specific autoimmune disorder. While eating disorders have been associated with other autoimmune diseases, no studies have explored this relationship in patients with HT to date. This study aimed to evaluate eating behaviors in euthyroid patients with HT.

Materials and methods

This case-control, cross-sectional study included a total of 107 patients diagnosed with HT, aged 18-45 years, as well as 54 healthy volunteers. Thyroid function tests, anti-thyroid peroxidase (TPO), and anti-antithyroglobulin (Tg) antibodies were measured in all participants. The Three-Factor Eating Questionnaire (TFEQ), the Night Eating Questionnaire (NEQ), and the International Physical Activity Questionnaire (IPAQ) were used to assess eating disorders and physical activity levels.

Results

Compared to the healthy control group, patients with HT, especially those receiving levothyroxine (LT4), had significantly higher scores on the TFEQ and NEQ. A positive correlation was detected between TFEQ and NEQ scores and anti-TPO and anti-Tg levels, LT4 treatment duration, and LT4 dose. However, there were no significant differences between the groups in terms of the IPAQ scores. A negative correlation was found between TFEQ scores and serum triiodothyronine (sT3). In patients with HT, thyroid-stimulating hormone (TSH) levels were within the higher-normal range, while sT3 levels were lower-normal compared to controls.

Conclusion

There is a spectrum of eating disorders among patients with HT. The underlying cause of these disorders remains unclear and may be associated with thyroid antibodies and/or hormonal status. In LT4 replacement therapy, efforts should be made to mimic true physiology as closely as possible. In the follow-up of patients with HT, while TSH is within lower-normal limits, sT3 levels may be observed to be in a higher-normal range. In selected cases, a combination of T4/T3 therapy or T3 extract may be recommended. Close monitoring of patients with HT is essential, particularly for potential eating disorder-related complications, such as obesity.

## Introduction

Hashimoto’s thyroiditis (HT) is an organ-specific autoimmune disorder and the leading cause of primary hypothyroidism in iodine-sufficient regions [1]. HT is a chronic inflammatory condition characterized by the infiltration of thyroid parenchyma by T cells, leading to varying degrees of thyroid dysfunction, accompanied by destruction and fibrosis of the gland [2].

To date, there has been limited research on the relationship between autoimmune diseases and eating disorders. Studies have identified eating disorders in patients with autoimmune conditions such as type 1 diabetes mellitus [3], Crohn’s disease [4], celiac disease [5], and psoriasis [6]. It has been hypothesized that these autoimmune diseases affect eating behaviors through neuroimmunoendocrine pathways. Various factors can influence eating attitudes and behaviors, including somatic diseases, hormones, genetics, environment, emotional state, sociodemographic characteristics, past experiences, cultural and religious beliefs, media, body image, obesity, and appetite. Eating behaviors are typically classified as healthy eating, external eating, restrictive eating, excessive eating, and emotional eating while eating disorders are categorized as anorexia nervosa (AN), bulimia nervosa (BN), binge eating disorder, pica, rumination disorder, and avoidant/restrictive food intake disorder [7].

In abnormal thyroid function states (hypothyroidism and hyperthyroidism), disturbances in eating and nutritional behaviors are expected. However, the relationship between eating disorders and thyroid function tests (TFT) in euthyroid patients remains unknown. To the best of our knowledge, no studies in the literature have examined the connection between HT and eating disorders. Therefore, the current study will be the first of its kind. Our hypothesis is that, even in a euthyroid state, inflammatory and immunological mechanisms in patients with HT may lead to disruptions in eating attitudes and behaviors. Furthermore, the degree of these disruptions may be related to thyroid autoantibody levels and TFTs, which remain within the normal range in these patients. This study aimed to compare the eating attitudes and behaviors of euthyroid patients with HT to those of a healthy control group.

## Materials and methods

Study sample 

This single-center, case-control, cross-sectional study was conducted at the Endocrinology and Metabolism Clinic of the University of Health Sciences Bursa Yuksek Ihtisas Training and Research Hospital between February 2021 and June 2024. A total of 107 individuals aged 18-45 years diagnosed with HT who consecutively presented to the clinic, as well as 54 healthy volunteers, were included in the study. The healthy control group was matched with age- and gender-matched individuals with negative thyroid autoantibodies who did not use medication and had no comorbidities who applied to our hospital for check-ups. 

Study protocol 

Patients who were under follow-up with a confirmed diagnosis of HT or were newly diagnosed with HT but had normal TFT values were enrolled. The exclusion criteria were as follows: 1) pregnancy and lactation, 2) a diagnosis of type 1 or type 2 diabetes mellitus, 3) hypertension and/or use of antihypertensive medications, 4) dyslipidemia and/or use of lipid-lowering drugs, 5) acute or chronic liver and kidney disease, 6) a history of malignancy, 7) additional autoimmune diseases, 8) being unwilling to participate or having mental retardation, psychiatric conditions (eating disorder, i.e., AN or bulimia) or inability to read and respond to the questionnaires.

Demographic data, including age, gender, educational level, marital status, alcohol and smoking habits, levothyroxine (LT4) usage (dose in mcg/day and duration in years), and other sociodemographic characteristics, were recorded. All patients underwent a physical examination. Body weight (kg) and height (cm) were measured, and body mass index (BMI) was calculated (kg/m²). Blood samples from all participants were collected between 08:00 and 09:00 following an eight-hour fast. Thyroid-stimulating hormone (TSH), free thyroxine (sT4), free triiodothyronine (sT3), anti-thyroid peroxidase (anti-TPO), and anti-thyroglobulin (anti-Tg) parameters were analyzed. Patients were considered to have biochemical HT if either anti-TPO was ≥45 IU/mL or anti-Tg was ≥45 IU/mL (reference ranges: TSH, 0.35-4.94 uIU/mL; sT4, 0.7-1.48 ng/dL; sT3, 1.58-3.91 pg/mL; anti-TPO, 0-5.61 IU/mL; and anti-Tg, 0-4.11 IU/mL). 

Data collection tools 

Three-Factor Eating Questionnaire (TFEQ)

This scale assesses eating behaviors based on three factors: cognitive restraint, disinhibition, and hunger. It measures the degree to which individuals consciously restrict food intake, their tendency toward uncontrolled eating, and their propensity to eat during emotional states. Originally developed by Stunkard and Messick with 51 items, the scale was adapted to Turkish culture by Karavuş et al. and finalized with 18 items. The scale is scored on a four-point Likert scale, with higher scores indicating greater levels of the above-mentioned eating behaviors [7,8]. 

Night Eating Questionnaire (NEQ)

Developed by Allison et al., the NEQ was adapted into Turkish by Atasoy et al. in 2014. The 14-item questionnaire evaluates eating patterns, including the timing of first food intake, morning appetite, evening and nighttime eating, food consumption after dinner, control over nighttime eating, difficulty falling asleep, frequency of waking up to eat, awareness of nighttime eating, and mood. Higher scores indicate a greater risk for night eating syndrome [9]. 

International Physical Activity Questionnaire (IPAQ)

This questionnaire was developed by the World Health Organization to assess physical activity levels across countries, and the reliability and validity analyses of the Turkish version of the scale were undertaken by Öztürk in 2005. When administering the IPAQ, physical activities were considered based on the criterion that they must be performed for a minimum of 10 minutes. Using this questionnaire, the participants were asked about the duration (in minutes) of the following activities over the past seven days: vigorous physical activities (e.g., football, basketball, aerobics, fast cycling, weightlifting, and carrying heavy loads), moderate physical activities (e.g., light load carrying, cycling at a normal pace, folk dancing, dancing, bowling, and table tennis), and walking and daily sitting times. Physical activity times were converted to metabolic equivalent task (MET), and total physical activity scores were calculated in MET minutes/week [10]. 

Prior to the study, ethical approval was obtained from the Ethics Committee of Bursa Yuksek Ihtisas Training and Research Hospital (approval number: 2011-KAEK-25 2022/01-08). All participants were informed about the study before participation. All procedures in this study were conducted in accordance with the ethical standards of institutional and/or national research committees and the 1964 Declaration of Helsinki and its later amendments or comparable ethical standards.

Statistical analysis 

After the data obtained from the research was coded, it was transferred to the computer to be analyzed using the Statistical Package for the Social Sciences (IBM SPSS Statistics for Windows, IBM Corp., Version 22, Armonk, NY) package program. In statistical analyses, the suitability of all continuous variables for normal distribution was evaluated using the Shapiro-Wilk test. While continuous data were expressed as mean ± standard deviation and median (minimum-maximum) values, categorical data were described using numbers and percentages. Categorical data between groups were evaluated using the Pearson chi-square or Fisher’s exact test in accordance with the expected value rule. Since it was determined that continuous variables did not follow a normal distribution, comparisons between the two groups were undertaken using the Mann-Whitney U test. In all statistical comparisons, the statistical significance level was accepted as p < 0.05.

## Results

The study included a total of 161 volunteers, comprising 107 (66.5%) patients with HT and 54 (33.5%) healthy controls. Women constituted 94.4% of the patients in the HT group and 87% of those in the control group, and there were no statistically significant differences between the groups in terms of gender distribution or median age (p = 0.129 and p = 0.923, respectively). 

In terms of eating habits, 62.6% of the patients in the HT group had two meals per day, whereas 51.9% of the controls had three meals per day. In addition, 52.3% of the patients with HT and 61.1% of the controls reported consuming snacks. However, no statistically significant difference was found between the groups regarding meal frequency or snack consumption (p = 0.079 and p = 0.290, respectively). Furthermore, while 39.3% of the patients in the HT group and 50.0% of those in the control group did not skip meals, a significant difference was found in meal-skipping patterns, with the control group more likely to skip dinner (p = 0.022). 

Of the patients with HT, 58.9% were receiving LT4 therapy, with an average LT4 dose of 104.6 ± 38.4 mcg/day (median = 100.0 mcg/day, range: 20-200 mcg/day). When comparing clinical, hormonal, and biochemical values, the median levels of TSH, anti-TPO, anti-Tg, glucose, triglycerides, insulin, and homeostatic model assessment for insulin resistance score were significantly higher in the HT group (p < 0.05 for all). In contrast, the median sT3 level was statistically higher in the control group (p = 0.008). Table 1 presents the detailed comparisons of hormonal, biochemical, and sociodemographic characteristics.

**Table 1:** Clinical hormonal and biochemical characteristics of patients with Hashimoto’s thyroiditis and healthy controls Anti-Tg: anti-thyroglobulin, anti-TPO: anti-thyroid peroxidase, HbA1c: hemoglobin A1c, HDL: high-density lipoprotein, HOMA-IR: homeostatic model assessment of insulin resistance, LDL: low-density lipoprotein, LT4: levothyroxine, sT3: free triiodothyronine, sT4: free thyroxine, TSH: thyroid-stimulating hormone ^#^Mann-Whitney U value; ^£^chi-square value; ^¥^t value (t-test)

Variables		Hashimoto’s thyroiditis group (n = 107)	Healthy control group (n = 54)	p-value	U/t/χ² value
Age (years)	Mean ± SD	41.1 ± 9.4	40.7 ± 9.9	0.923	2862.0^£^
	Median (min-max)	41.0 (18-59)	42.0 (22-59)		
Gender, n (%)	Female	101 (94.4)	47 (87.0)	0.129	2.616^£^
	Male	6 (5.6)	7 (13.0)		
Height (cm)	Mean ± SD	161.8 ± 7.6	163.8 ± 6.5	0.655	2713.0^#^
	Median (min-max)	160.0 (149-197)	163.5 (150-180)		
Body weight (kg)	Mean ± SD	76.0 ± 17.4	69.7 ± 11.4	0.039	2269.5^#^
	Median (min-max)	75.0 (50-135)	69.0 (50-94.8)		
BMI (kg/m^2^)	Mean ± SD	29.1 ± 6.2	25.9 ± 3.9	0.003	2050.0^#^
	Median (min-max)	28.8 (18.9-49.4)	24.8 (19.2-35.9)		
Educational level, n (%)	Illiterate	4 (3.7)	0 (0.0)	0.054	9.28^£^
	Primary school	31 (29.0)	8 (14.8)		
	Middle school	8 (7.5)	10 (18.5)		
	High school	28 (26.2)	17 (31.5)		
	University	36 (33.6)	19 (35.2)		
Marital status, n (%)	Single	12 (11.2)	14 (25.9)	0.017	5.73^£^
	Married	95 (88.8)	40 (74.1)		
Visiting a dietician, n (%)	Yes	46 (43.0)	11 (20.4)	0.005	8.78^£^
	No	61 (57.0)	43 (79.6)		
Meals per day, n (%)	2	67 (62.6)	26 (48.1)	0.079	2.69^£^
	3	40 (37.4)	28 (51.9)		
Snacking, n (%)	Present	56 (52.3)	33 (61.1)	0.290	1.36^£^
	Absent	51 (47.7)	21 (38.9)		
Skipped meals, n (%)	Breakfast	23 (21.5)^a*^	9 (16.7)^a*^	0.022	11.45^£^
	Lunch	40 (37.4)^a*^	12 (22.2)^a*^		
	Dinner	0 (0.0)^a*^	3 (5.6)^b*^		
	Variable	2 (1.9)^a*^	3 (5.6)^a*^		
	None	42 (39.3)^a*^	27 (50.0)^a*^		
Smoking status, n (%)	Never	72 (67.3)	37 (68.5)	0.950	0.10^£^
	Past	13 (12.1)	7 (13.0)		
	Current	22 (20.6)	10 (18.5)		
Duration of Hashimoto’s thyroiditis diagnosis (years)	Mean ± SD	7.95 ± 6.02	-		
	Median (min-max)	6.0 (1-30)			
LT4 use (present/absent)	n1/n2	63/44	0/54	<0.001	52.23^£^
LT4 dose (mcg/day)	Mean ± SD	104.6 ± 38.4	-		
	Median (min-max)	100.0 (20-200)			
TSH	Mean ± SD	2.49 ± 1.49	1.76 ± 1.10	0.004	2078.5^#^
	Median (min-max)	2.32 (0.16-5.77)	1.38 (0.45-4.63)		
sT4	Mean ± SD	1.01 ± 0.22	1.00 ± 0.15	0.798	2817.5^#^
	Median (min-max)	0.97 (0.67-2.50)	0.98 (0.79-1.65)		
sT3	Mean ± SD	2.70 ± 0.43	2.86 ± 0.52	0.008	2148.0^#^
	Median (min-max)	2.68 (0.87-3.75)	2.86 (0.91-3.71)		
Anti-TPO	Mean ± SD	451.8 ± 370.9	0.88 ± 1.00	<0.001	111.5^#^
	Median (min-max)	355.0 (0.51-1000)	0.55 (0.0-4.72)		
Anti-Tg	Mean ± SD	185.14 ± 305.21	2.08 ± 3.50	<0.001	253.0^#^
	Median (min-max)	49.18 (0.1-1000)	0.96 (0.11-22.12)		
Fasting blood glucose	Mean ± SD	92.7 ± 11.9	85.3 ± 8.1	<0.001	1874.5^#^
	Median (min-max)	90.0 (56-126)	87.0 (69-99)		
LDL	Mean ± SD	120.9 ± 35.7	113.0 ± 28.8	0.144	3.11^¥^
	Median (min-max)	123.2 (45.7-206.7)	109.1 (67.0-176.0)		
Total cholesterol	Mean ± SD	198.3 ± 44.5	189.2 ± 35.9	0.167	2.23^¥^
	Median (min-max)	198.0 (73.6-372.0)	191.0 (87.9-272.0)		
HDL	Mean ± SD	55.4 ± 14.4	57.6 ± 9.88	0.120	10.88^¥^
	Median (min-max)	53.7(30.5-101.7)	57.8 (36.0-77.0)		
TG	Mean ± SD	121.0 ± 66.8	95.4 ± 47.2	0.012	2190.5^#^
	Median (min-max)	103.0(16-393)	89.5 (10.3-265.0)		
HBA1c	Mean ± SD	5.31 ± 0.50	5.25 ± 0.42	0.661	2766.5^#^
	Median (min-max)	5.29 (4.09-6.90)	5.29 (4.49-6.09)		
Insulin	Mean ± SD	9.95 ± 6.01	6.62 ± 2.43	<0.001	1804.0^#^
	Median (min-max)	8.80 (3.0-35.0)	6.15 (2.6-13.9)		
HOMA-IR	Mean ± SD	2.30 ± 1.50	1.41 ± 0.57	<0.001	1692.5^#^
	Median (min-max)	1.97 (0.64-9.07)	1.31 (0.48-3.26)		

Concerning the nutritional scales used in the study, the median scores for the TFEQ and NEQ were significantly higher in the HT group compared to the control group (p < 0.001 for all). However, no statistically significant difference was found between the groups’ IPAQ scores (p = 0.824). In addition, the patients with HT who were receiving LT4 treatment had significantly higher median TFEQ and NEQ scores (p < 0.001 and p = 0.007, respectively). Further details are provided in Table 2. 

**Table 2:** Comparison of questionnaire scores between the study groups HT: Hashimoto’s thyroiditis, LT4: levothyroxine ^#^Mann-Whitney U value; ^¥^t value

Variables	HT group (n = 107)	HC group (n = 54)	p, t/U value	LT4 (+) HT (n = 63)	LT4 (-) HT (n = 98)	p
Three-Factor Eating Questionnaire	40.7 ± 8.4	31.9 ± 11.2	<0.001, 3.015^¥ ^	41.2 ± 8.4	35.5 ± 10.8	<0.001, 2.791^¥ ^
	39 (25-64)	31 (18-63)		40 (25-64)	34.5(18-63)	
Night Eating Questionnaire	16.4 ± 7.6	10.4 ± 5.5	<0.001, 1,361.5^# ^	16.4 ± 8.0	13.1 ± 6.9	0.007, 2,311.0^# ^
	14 (2-43)	9 (4-27)		14 (2-43)	12 (2-36)	
International Physical Activity Questionnaire	1,450.0 ± 1,504.9	1,315.6 ± 1,049.73	0.824, 2,827.0^#^	1,344.2 ± 1,363.9	1,444.0 ± 1,374.8	0.395, 2,841.5^# ^
	900 (247-9,093)	924 (99-4,605)		882.0 (247.5-6,984.0)	924.0 (99.0-9,093.0)	

Correlation analyses were performed to evaluate the relationships between the nutritional scale scores and certain hormonal, biochemical, and clinical parameters. A weak level of significant negative correlation was found between TFEQ scores and sT3 levels (r = -0.17, p = 0.026). Moderate levels of significant correlations were observed between TFEQ scores and anti-TPO, anti-Tg levels, LT4 treatment duration, and LT4 dose (r = 0.29, p < 0.001; r = 0.25, p = 0.001; r = 0.28, p < 0.001; and r = 0.26, p = 0.001, respectively). Similarly, NEQ scores were significantly correlated with TSH, anti-TPO, and anti-Tg levels, as well as LT4 treatment duration and dosage (r = 0.17, p = 0.036; r = 0.33, p < 0.001; r = 0.35, p < 0.001; r = 0.23, p = 0.003; and r = 0.22, p = 0.005, respectively). Further details are provided in Table 3.

**Table 3:** Correlation between questionnaire scores and various parameters Anti-Tg: anti-thyroglobulin, anti-TPO: anti-thyroid peroxidase, LT4: levothyroxine, sT3: free triiodothyronine, sT4: free thyroxine, TSH: thyroid-stimulating hormone *Correlation coefficient (r value), **p-value

Variables	TSH	sT4	sT3	Anti-TPO	Anti-Tg	LT4 treatment duration	LT4 dose
Three-Factor Eating Questionnaire	0.13*	-0.05*	-0.17*	0.29*	0.25*	0.28*	0.26*
	0.09**	0.95**	0.026**	<0.001**	0.001**	<0.001**	0.001**
Night Eating Questionnaire	0.17*	0.12*	-0.04*	0.33*	0.35*	0.23*	0.22*
	0.036**	0.12**	0.61**	<0.001**	<0.001**	0.003**	0.005**
International Physical Activity Questionnaire	-0.13*	0.03*	0.02*	-0.07*	-0.02*	-0.04*	-0.06*
	0.08**	0.66**	0.84**	0.41**	0.71**	0.53**	0.42**

## Discussion

This is the first study to compare the eating behaviors of euthyroid patients with HT to healthy controls while also examining the relationship between these eating behaviors and thyroid autoantibodies, TFT levels, and LT4 replacement therapy in treated patients. The results revealed that euthyroid patients with HT, even those on LT4 therapy, had worse eating behavior questionnaire results compared to healthy controls. A positive correlation was observed between TFEQ and NEQ scores and anti-TPO and anti-Tg levels, LT4 treatment duration, and LT4 dose; however, physical activity scores were similar between the groups. 

Previous research on the relationship between autoimmune diseases and eating disorders has been limited, focusing mainly on conditions such as type 1 diabetes mellitus, Crohn’s disease, celiac disease, and psoriasis. These studies have consistently shown a bidirectional relationship between autoimmune diseases and eating disorders [3-6,11]. Autoimmune diseases are often accompanied by psychiatric disorders, including schizophrenia, mood disorders, and attention-deficit hyperactivity disorder [12]. Autoantibodies present in autoimmune conditions may cross-react with neuronal antigens (brain-reactive autoantibodies), potentially playing a role in the pathogenesis of neuropsychiatric diseases. A typical example is the post-streptococcal AN, where antineuronal antibodies have been detected [13]. Blanchin et al. demonstrated that anti-TPO antibodies could bind to human cerebellar astrocytes [14], suggesting that these antibodies could affect the brain and nervous system. However, the specific inflammatory and immunological markers involved in eating disorders are not yet well known. Some researchers have found antibodies against alpha-melanocyte-stimulating hormone (α-MSH), which regulates appetite and body weight, and adrenocorticotropic hormone (ACTH) in autoimmune diseases [15,16]. Researchers have specifically found a correlation between the psychobehavioral aspects and severity of eating behavior with the levels of autoantibodies against α-MSH [17]. However, there is limited information on the relationship between eating behaviors and HT. Based on the aforementioned mechanisms, the potential effect of thyroid antibodies on appetite and eating behavior in patients with HT has not yet been investigated. To our knowledge, the current study is the first to scientifically reveal the relationship between nutritional behaviors and anti-TPO and anti-Tg levels, offering a new perspective on monitoring autoantibodies in individuals with poor eating habits. 

In this study, higher eating behavior scores were observed in patients with HT, particularly those on LT4 therapy, compared to controls. Until now, it was believed that normal TSH levels were accepted to indicate proper and adequate treatment for patients [18]. However, these results suggest that exogenous LT4 replacement may not fully mimic normal physiology, especially as higher questionnaire scores were found to be associated with longer duration and higher doses of LT4 treatment. The target TSH level in exogenous LT4 replacement remains a subject of debate [19] As is known, higher-normal TSH levels are targeted in elderly patients and those with a history of cardiovascular disease or osteoporosis, while lower-normal TSH is targeted in younger patients and those with dyslipidemia [20]. However, there is no established TSH range for other clinical factors such as BMI, fat mass, mood, quality of life, or cognitive function. As indicated by the results of the current study, higher-normal TSH may be associated with eating disorders. Therefore, we believe that a specific TSH target should be established for patients with HT. 

Although patients treated with LT4 are euthyroid, symptoms resembling hypothyroidism, such as fatigue, mood disturbances, body pain, and exercise intolerance, often persist [21,22]. This suggests the possibility of hypothyroidism at the tissue or cellular level. Serum TSH remains the best available marker for assessing adequate thyroid replacement, but due to possible discordance between TSH and tissue or cellular thyroid function, the most accurate measure of euthyroidism remains tissue biopsy and gene expression analysis. However, these methods are not practical for routine clinical use. Therefore, studies have explored the use of triiodothyronine extract or LT4/LT3 combinations [23,24]. In our study, the lower sT3 levels in patients with HT compared to controls suggest that TE or LT4/LT3 combinations may be beneficial for certain patients, even when TSH is within the normal range. The larger longitudinal interventional studies are needed. 

Previous studies have revealed a bidirectional relationship between obesity and thyroid autoimmunity [25]. The primary mechanisms implicated in this relationship include various metabolic factors secreted by white adipose tissue, known as adipokines (resistin and visfatin), along with proinflammatory cytokines such as interleukin (IL)-6, IL-12, and tumor necrosis factor-alpha. The pro-inflammatory process negatively affects eating behavior, exacerbating existing obesity, while increased obesity, in turn, leads to elevated antibody levels, creating a vicious cycle. Considering the results of our study, it is suggested that patients with HT may have more prevalent eating disorders, which, if not recognized or assessed from this perspective, could further contribute to their obesity. 

HT is the most common cause of primary hypothyroidism, and its incidence has increased in recent years. This rise is largely attributed to genetic predispositions, alongside factors such as industrialization, air pollution, urbanization, personal hygiene, socioeconomic status, psychological stress, sedentary lifestyles, and dietary habits. In particular, the increasing consumption of high-calorie, high-fat, high-sugar, and low-fiber foods, a characteristic of the “Western diet,” directly disrupts immune system balance and intestinal microbiota and increases inflammation, leading to an increase in the prevalence of HT and other autoimmune diseases such as type 1 diabetes mellitus, rheumatoid arthritis, and multiple sclerosis, as well as obesity through an increase in fat mass [26]. These patients are encouraged to adopt a Mediterranean diet, which includes vegetables, fresh fruits, olive oil, fish, nuts, whole grains, and lower amounts of processed and red meat. Due to its anti-inflammatory and antioxidant properties, the Mediterranean diet is believed to have immunomodulatory effects that positively influence gut microbiota and cytokine production, offering protection against inflammatory and autoimmune diseases [27-29]. In cases of both poor food choices and eating disorders, the onset of obesity is an expected outcome. Therefore, a multidisciplinary approach to obesity is essential, involving a team of dietitians, endocrinologists, internal medicine specialists, and physiotherapists. Including a psychiatrist and/or psychologist in this team is especially important. 

The strength of this study lies in its focus on the relationship between eating disorders and thyroid autoantibodies in patients with HT, even in the absence of abnormal TSH levels. It is also the first study to investigate the relationship between eating disorders, TFT levels, and thyroid autoantibodies in euthyroid patients with HT. Limitations of the study include the small sample size, single-center design, and reliance on self-reported data. Multicenter design, objective behavioral assessments larger longitudinal interventional studies are needed. 

## Conclusions

In conclusion, eating disorders have an increased prevalence among patients with HT, although their exact etiology remains unclear. This issue is particularly prominent in those receiving LT4 replacement therapy. The optimal TSH target level for LT4 replacement therapy remains a matter of debate. Personalized treatment goals should be established, not only for patients with HT but for all individuals undergoing LT4 therapy, and unnecessary operations should be avoided. Patients with HT should be evaluated in terms of appetite, nighttime eating issues, dietary habits, food intake, physical activity, and weight gain. Further multicenter, multidisciplinary, and prospective studies with larger sample sizes are needed in this area.
